# Administration with corticosteroid relieving pain following total knee arthroplasty: A meta-analysis

**DOI:** 10.1097/MD.0000000000023567

**Published:** 2020-12-18

**Authors:** Jiao Zhang, Ji-xun Huang

**Affiliations:** a pharmaceutical department; b deputy director of Jingjiang people's hospital, Jingjiang, Tai Zhou city, Jiangsu province, China.

**Keywords:** analgesia, corticosteroid, meta-analysis, total knee arthroplasty

## Abstract

**Background:**

This meta-analysis compares the effectiveness of corticosteroid in relieving pain and inflammation in total knee arthroplasty (TKA) patients.

**Method:**

Randomized controlled trials in PubMed (1996 to March 2020), Embase (1996 to March 2020), and the Cochrane Library (CENTRAL, March 2020) compared corticosteroid and placebo in pain in TKA patients were identified by a software and manual searching. The risk of bias and clinical relevance of the included studies were assessed. Sensitivity analysis was performed by omitting each study in turn. The major outcomes of the studies were analyzed by the Stata 12.0.

**Results:**

13 randomized controlled trials that involved 193 patients were included in the present meta-analysis. The results of the study revealed a significantly lower visual analog scale (VAS) score of pain at rest in the corticosteroid group (12 hours: weighted mean difference (WMD)=−1.35, *P* = .005; 24 hours: WMD=−1.11, *P* = .000; 48 hours: WMD=−0.31, *P* = .000; 72 hours: WMD = −0.30, *P* = .000). And Postoperative VAS scores during mobilization at 12 hours and 24  hours were significantly lower at corticosteroid group when compared with control group (12 hours: WMD = −0.81, P = 0.000; 24 hours: WMD = −1.66, *P* = .018). Meta-analyses show that administration of corticosteroid can reduce the length of hospital stay, incidence nausea and the C-reactive protein level. While no significant difference was observed in the VAS scores during mobilization at 48 hours and 72 hours and total morphine consumption (*P* > .05).

**Conclusions:**

Compared to the control group, intraoperative corticosteroid was benefit to the pain management in TKA. However, more high-quality studies are still warranted to further validate our findings, considering there are several limitations in this meta-analysis.

## 1 Introduction

Total knee arthroplasty (TKA) is 1 of the most effective methods for end-stage of knee osteoarthritis (OA) and improve the quality of life of patients.^[1,2]^ According to epidemiological statistics of OA, the incidence of OA in people over 65 years and over 80 years old can reach 50% and 80% respectively.^[3]^ Over 90 000 TKAs are performed annually in England and Wales.^[4]^ However, there are some problems to be solved after TKA.

Patients frequently experience postoperative pain after a TKA; such pain is always challenging to treat and may delay the patients recovery.^[5]^ Inadequate management of postoperative pain results in increased morbidity, delayed discharge, and decreased patient satisfaction after TKA. Therefore, adequate pain relief following TKA can promote early rehabilitation and increase patients’ satisfaction.^[6]^ Corticosteroid has strong anti-inflammatory properties and relieve pain following surgeries.^[7]^ Recently several published studies demonstrated the superiority of corticosteroid in analgesic effect compared to the non-corticosteroid group.^[8]^ There is a growing consensus that the corticosteroid should be recommended as the analgesic choice for patients undergoing TKA. While the necessary to use corticosteroid was remaining controversy.

In light of the ongoing controversy about the application of corticosteroid in TKA, we have conducted this meta-analysis to assess the effect of corticosteroid on pain intensity and morphine consumption, and subsequently inform the design of future studies to help definitively address these areas.

## 2 Materials and methods

This meta-analysis was performed in accordance with the PRISMA checklist (Preferred Reporting Items for Systematic Reviews and Meta-Analyses). This article does not contain any studies with human participants or animals performed by any of the authors and no ethical review was need for this meta-analysis.

### 2.1 Search strategy

**Figure 1:**
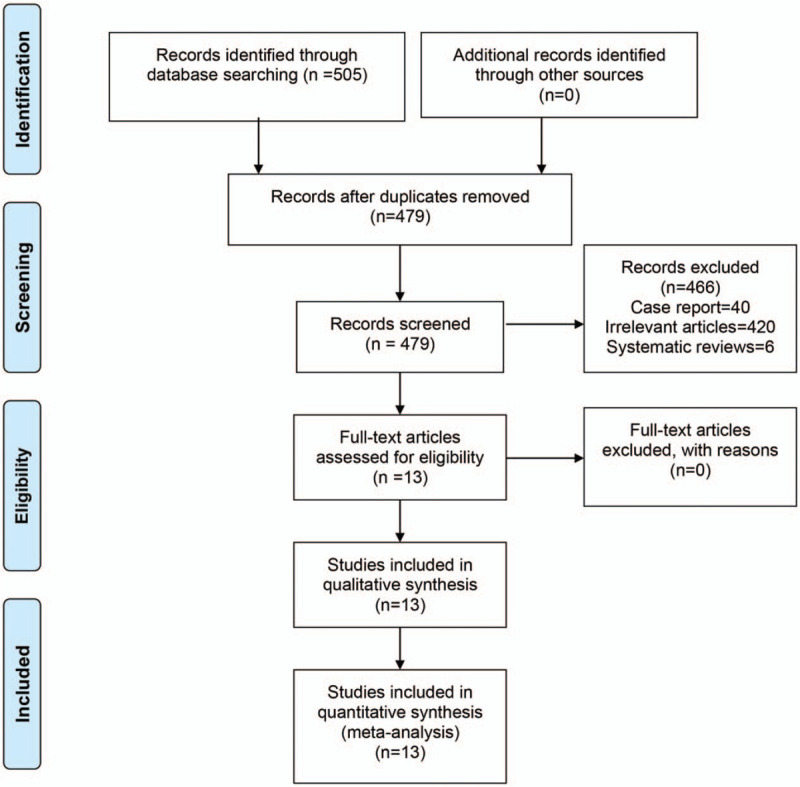
Flow diagram of article screening process.

We systematically searched PubMed (1996 to March 2020), Embase (1996 to March 2020), and the Cochrane Library (CENTRAL, March 2020). To identify trials that may not have been published in full or were missed through the electronic search, we manually searched all references from the included studies and relevant previous systematic reviews. Search items were as follows: “Total knee arthroplasty”, “TKA”, “Total knee replacement,” “TKR,” “Arthroplasty, Replacement, Knee[Mesh],” “Dexamethasone[Mesh]” and “corticosteroid” were used as key words using Boolean operators ‘AND’ or ‘OR’. Flow diagram results are shown in Figure 1.

### 2.2 Inclusion and exclusion criteria

Trials were included in our meta-analysis on condition they met the PICOS (patients, intervention, comparator, outcome, study design) criteria.

(1) Patients: patients had received TKA for the first time.
(2) Intervention: corticosteroid for TKA.
(3) Comparator: non-corticosteroid
(4) Outcomes: visual analog scale (VAS) at rest,

VAS at movement, total morphine consumption, the occurrence of nausea, periprosthetic knee infection, length of stay and C-reactive protein (CRP) level. 5. Study design: randomized controlled trials (RCT).

### 2.3 Data extraction

Two researchers collected available data from included studies independently, and any disagreement between the 2 researches was judged by discussion. Basic characteristics including author, country, number of patients, age, female patients, body mass index, study, anesthesia, type of dexamethasone and follow-up. Morphine consumption was were converted to a uniform unit according the standard formula.^[9]^ Pain VAS scores (range, 0–10; a score of 0 indicated ‘no pain’ and higher scores indicate higher pain intensity) were extracted from all of the included studies. Secondary outcomes consisted of total morphine consumption, the occurrence of nausea, periprosthetic knee infection, length of hospital stay and CRP level.

### 2.4 Risk of bias assessment

The quality of the included RCTs was assessed by 2 investigators according to the Cochrane Collaboration's tool for assessing risk of bias. The assessment contains 7 items: randomization sequence generation, allocation concealment, blinding of participants and personnel, blinding of outcome assessment, incomplete outcome data, selective reporting and other bias. When there was more than one item with “unclear” or “high” risk of bias, the quality of the study was considered “unclear risk of bias” or “high risk of bias.” We will judge each component as being low risk of bias, high risk of bias or unclear risk of bias. All disagreements were resolved by the discussion.

### 2.5 Statistical analysis

The data referring to evaluations through VAS with rest or mobilization at 12 hours, 24 hours, 48 hours, and 72 hours, total morphine consumption, length of hospital stay was compared between groups of corticosteroid and control groups. As the data was reported with the mean value and the standard deviation, an exploratory meta-analysis would be conducted narratively using weighted mean difference (WMD) as the effect size. The heterogeneity was tested with *I*^2^, and in case of a significant heterogeneity (*I*^2^ > 50%), random-effect model and sensitivity analysis would be employed, while fixed-effect model would be selected. when presenting with excellent homogeneity. Funnel plot would be used to detect the existing publication bias. The statistical significance was defined at a 2-sided *P* value of < .05. The statistical procedures were conducted through software of Stata software (version 12.0, Stata Corp LLC, College Station, Texas).

## 3 Results

### 3.1 Search results

A total of 505 articles were identified, and their records were included in Endnote X7 (Clarivate Analytics, Philadelphia, PA). After removing 26 duplicates, remaining 479 articles were screened according to the titles and abstracts. A total of 466 articles were removed according to the inclusion criteria. Any disagreements about the inclusion of an article were resolved by the discussion. A full-text assessment was conducted on the rest of the 63 articles. Finally, 13 RCTs^[10–22]^ involving 1287 patients were finally included in this meta-analysis (corticosteroid = 651, control = 636). The basic characteristics and interventions are summarized in Table 1.

**Table 1:** General characteristic of the includes studies.

Author	Country	Number of patients	Age	Female patients	BMI	Study	Anesthesia	Type of dexamethasone	Follow-up
Xu 2018	China	60/61	64.5/65.8	82.5/85.9	25.6/28.8	RCT	Spinal anesthesia	Dexamethasone (10 mg)	3 months
Chia 2013	Australia	42/43	66.8/65.0	NS	31/31.4	RCT	Spinal anesthesia	Triamcinolone acetonide (80 mg)	12 weeks
Kim 2020	Korea	45/44	69.3/68.2	92.5/90.7	26.4/27.9	RCT	Spinal anesthesia	Dexamethasone (0.1mg/kg)	1 week
Koh 2013	Korea	135/134	72/72	87/89	26.3/26.1	RCT	Spinal anesthesia	Dexamethasone (10 mg)	4 weeks
Luna 2017	Denmark	21/19	68/67	71.4/42.1	28.8/28.2	RCT	Spinal anesthesia	Methylprednisolone 40 mg	1 month
Lunn 2011	Denmark	24/24	66/66	50/62	27/27	RCT	Spinal anesthesia	Methylprednisolone (125 mg)	1 month
Rytter 2017	Denmark	35/37	65/66	51.4/45.9	28.3/30.4	RCT	Spinal or general anesthesia	Methylprednisolone (125 mg)	1 month
Samona 2017	USA	55/47	64.8/62.6	54.5/59.6	NS	RCT	Spinal anesthesia or general anesthesia	Dexamethasone (8 mg)	48 hours
Tammachote 2020	Thailand	50/50	67/69	62.5/51.8	27/27	RCT	Spinal anesthesia	Methylprednisolone (40 mg)	72 hours
Tammachote 2018	Thailand	54/54	69/68	79.6/81.5	27/27	RCT	spinal anesthesia+ epidural anesthesia	Triamcinolone acetonide (40 mg)	3 months
Tsukada 2016	Japan	40/37	75/72	87.5/86.5	26.7/27.3	RCT	Spinal anesthesia	Methylprednisolone (40 mg)	7 days
Xu 2017	China	54/54	63.6/63.6	85.7/83.5	NS	RCT	Spinal anesthesia	Dexamethasone (10 mg)	3 days
Li 2019	China	36/32	63.9/64.7	80.6/84.3	25.3/24.7	RCT	Local infiltration anesthesia	Hydrocortisone (100 mg)	4 weeks

For these enrolled studies, the published years of them were ranged from 2011 to 2020. Regarding location where the studies were performed, 3 studies were from Denmark, 3 from China, 2 from Thailand, 2 from Korea, 1 from the USA, 1 from the Japan, and 1 from Australia. The number of cases enrolled in corticosteroid subjects ranged from 21 to 135 cases, and the number of control subjects varied from 19 to 134 subjects. The average age of the corticosteroid subjects and control subjects was 37.3 and 66.9 years respectively. The proportion of female patients ranged from 50% to 92.5%. body mass index in the corticosteroid subjects and control subjects was 27.2 and 27.8 respectively. All studies were RCTs. Nine studies use spinal anesthesia, 1 study use spinal or general anesthesia, 1 study use spinal anesthesia combined with epidural anesthesia and 1 study use local infiltration anesthesia. Follow-up duration ranged from 3 days to 3 months.

### 3.2 Risk of bias

**Figure 2:**
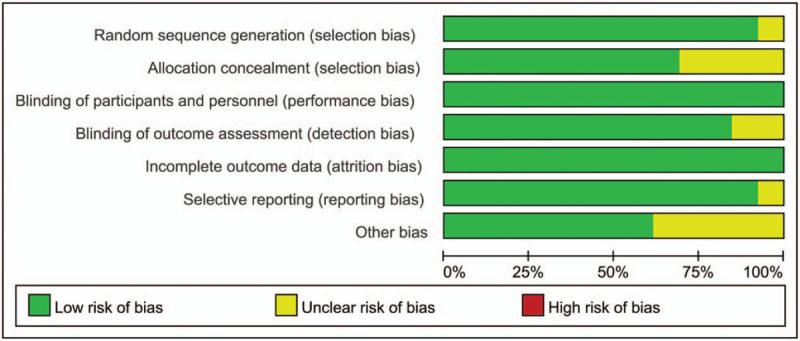
Risk of bias gragh.

**Figure 3:**
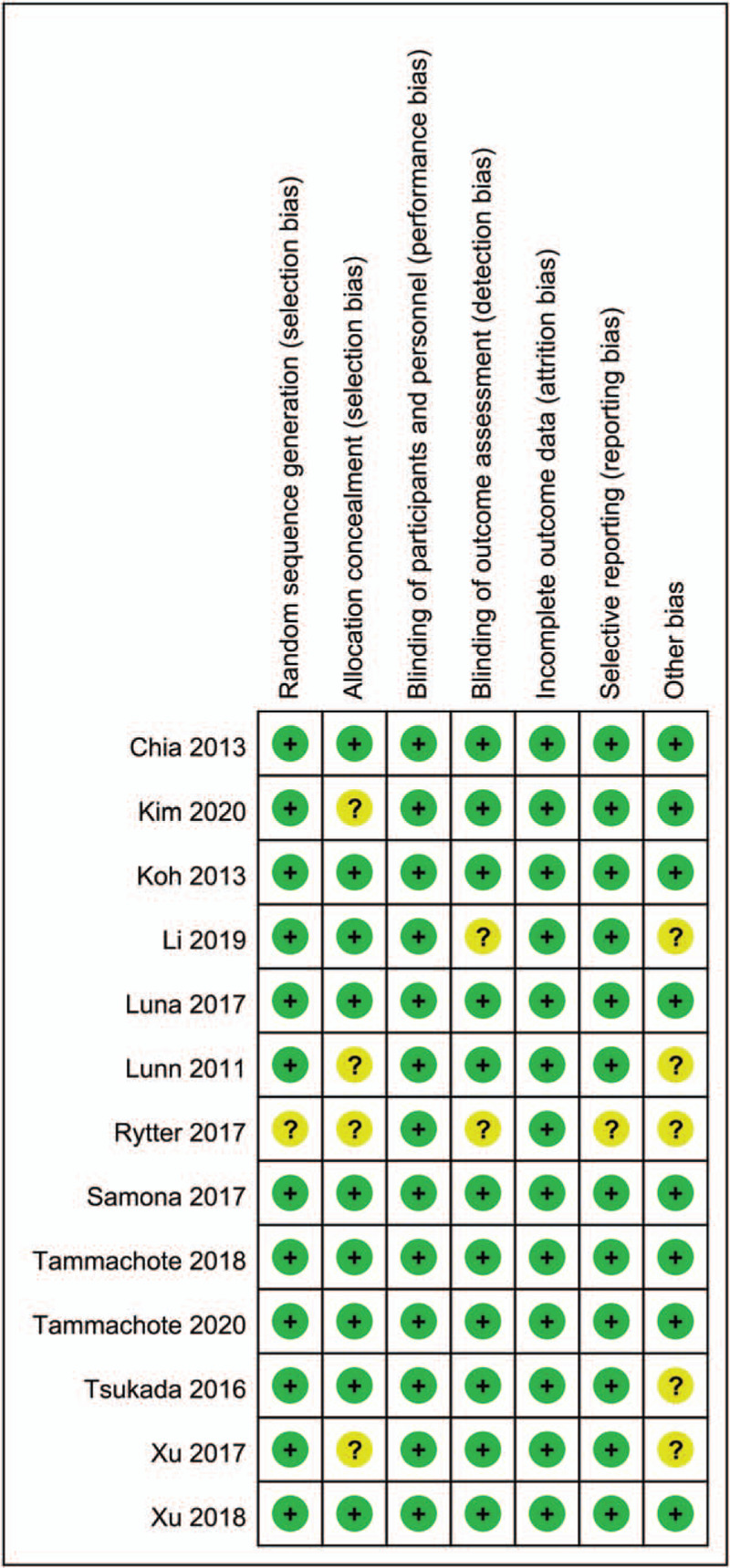
Risk of bias summary (“+”low risk;“?,” unclear risk;“−,” high risk).

The risk of bias summary and risk of bias graph of the thirteen included studies is summarized in Figures 2 and 3 respectively. Overall, study quality was relatively high, with 7 studies having low risk of bias, with 6 studies having unclear risk of bias and no studies with high risk of bias. Only one study was with unclear risk of bias for random sequence generation, and the other twelve studies as at low risk of bias because all perform right sequence generation. Four studies were with unclear risk of bias for allocation concealment. All of the studies were with low risk of bias for blinding of participants and personnel, all studies were with low risk of bias for blinding of outcome assessment. One study has unclear risk of bias for selective reporting bias. Five studies were with unclear risk of bias for other bias.

### 3.3 Results of meta-analysis

#### 3.3.1 VAS with rest

**Figure 4:**
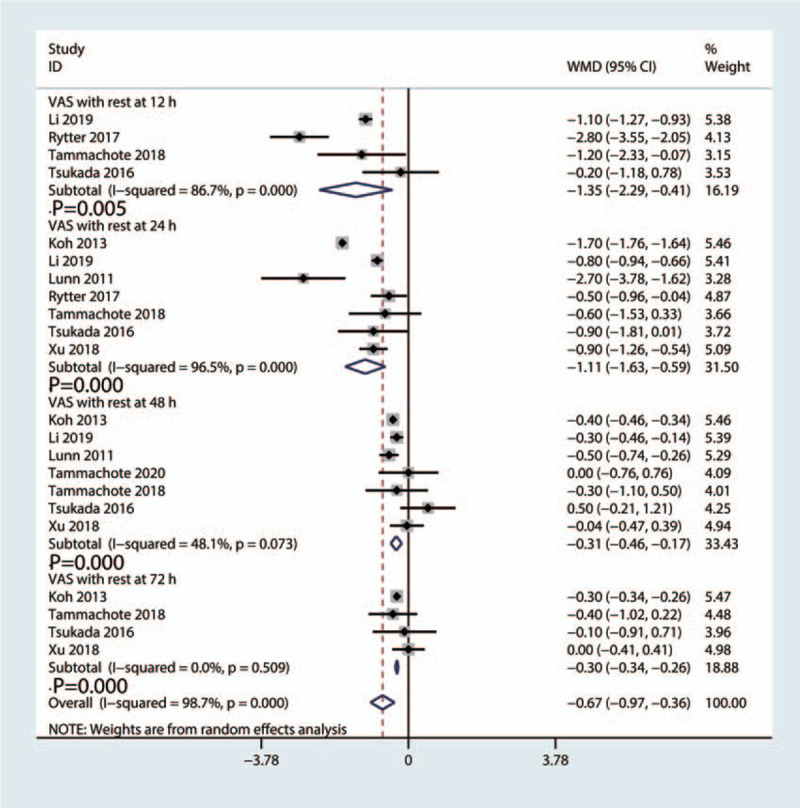
Forest plot of the comparison of VAS at rest at 12 hours, 24 hours, 48 hours and 72 hours between corticosteroid and control group.

Corticosteroid group had lower VAS at 12 hours (WMD = −1.35, 95%CI: [−2.29, −0.41], *P* = 0.005; *I*^2^ = 86.7%, *P*_heterogeneity_ = .000, Fig. 4), 24 hours (WMD = −1.11, 95%CI: [−1.63, −0.59], *P* = 0.000; *I*^2^ = 96.5%, *P*_heterogeneity_ = .000, Fig. 4), 48 hours (WMD = −0.31, 95%CI: [−0.46, −0.17], *P* = .000; *I*^*2*^ = 48.1%, *P*_heterogeneity_ = .073, Fig. 4), and 72 hours (WMD = −0.30, 95%CI: [−0.34, −0.26], *P* = .000; *I*^2^ = 0.0%, *P*_heterogeneity_ = .509, Fig. 4) when compared to the control group. A random effect model was used due to high heterogeneity in VAS at rest at 12 hours, 24 hours, 48 hours and 72 hours (*I*^2^ = 98.7%, *P* = .000, Fig. 4).

#### 3.3.2 VAS with mobilization

**Figure 5:**
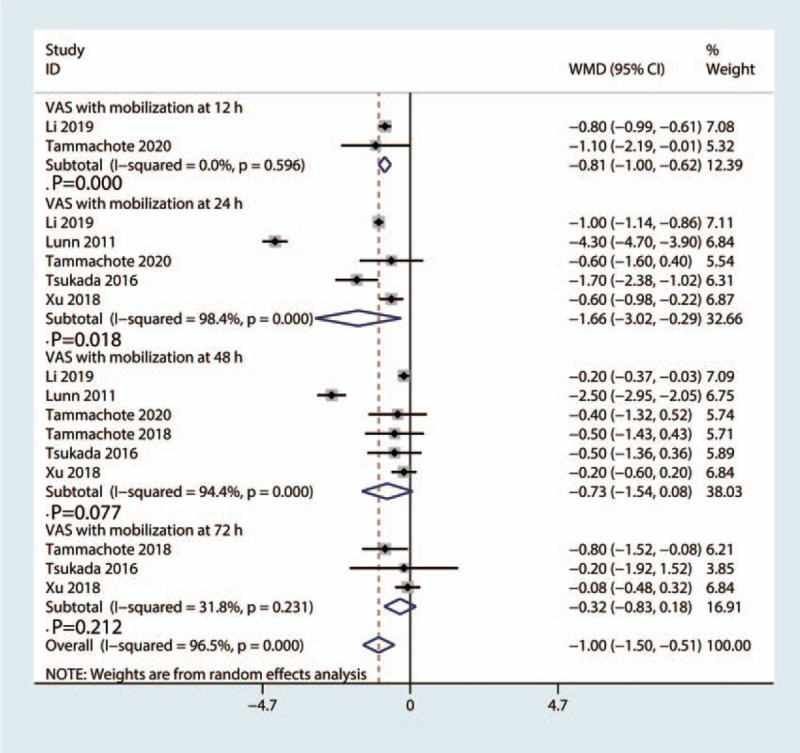
Forest plot of the comparison of VAS at mobilization at 12 hours, 24 hours, 48 hours and 72 hours between corticosteroid and control group.

Compared with the control group, the corticosteroid group showed lower VAS with mobilization at 12 hours (WMD = −0.81, 95%CI: [−1.00, −0.62], *P* = .000; *I*^2^ = 0.0%, *P*_heterogeneity_ = .596, Fig. 5), 24 hours (WMD = −1.66, 95%CI: [−3.02, −0.29], *P* = .018; *I*^2^ = 98.4%, *P*_heterogeneity_ = .000, Fig. 5), 48 hours (WMD = −0.73, 95%CI: [−1.54, 0.08], *P* = .077; *I*^2^ = 94.4%, *P*_heterogeneity_ = .000, Fig. 5); 72 hours (WMD = −0.32, 95%CI: [−0.83, 0.18], *P* = .212; *I*^2^ = 31.8%, *P*_heterogeneity_ = .231, Fig. 5).

#### 3.3.3 Total morphine consumption

**Figure 6:**
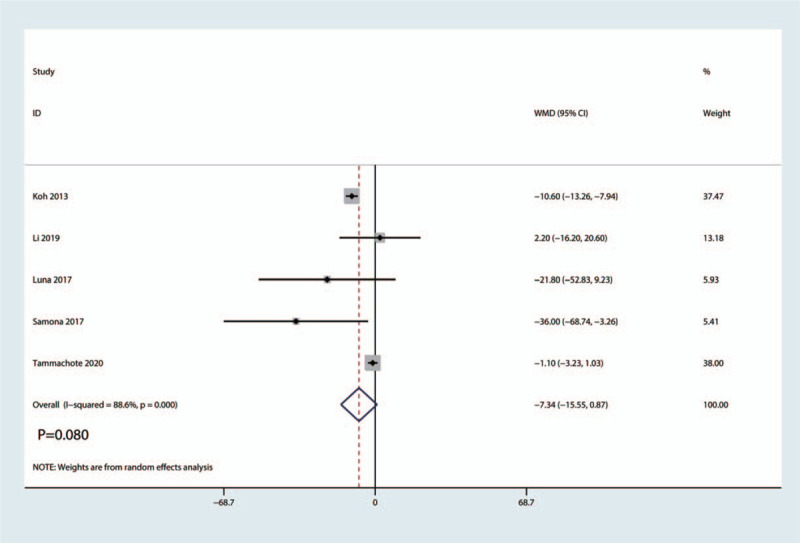
Forest plot of the comparison of total morphine consumption between corticosteroid and control group.

Data from 5 studies with 510 patients reported the total equivalent morphine consumption. Pooled data indicated that the corticosteroid group consumed less morphine compared to the control group (WMD = −7.34, 95%CI: [−15.55, 0.87], *P* = .080; Fig. 6). The random effects model was used because heterogeneity was significant (*I*^2^ = 88.6%, *P*_heterogeneity_ = .080; Fig. 6).

#### 3.3.4 Periprosthetic knee infection

**Figure 7:**
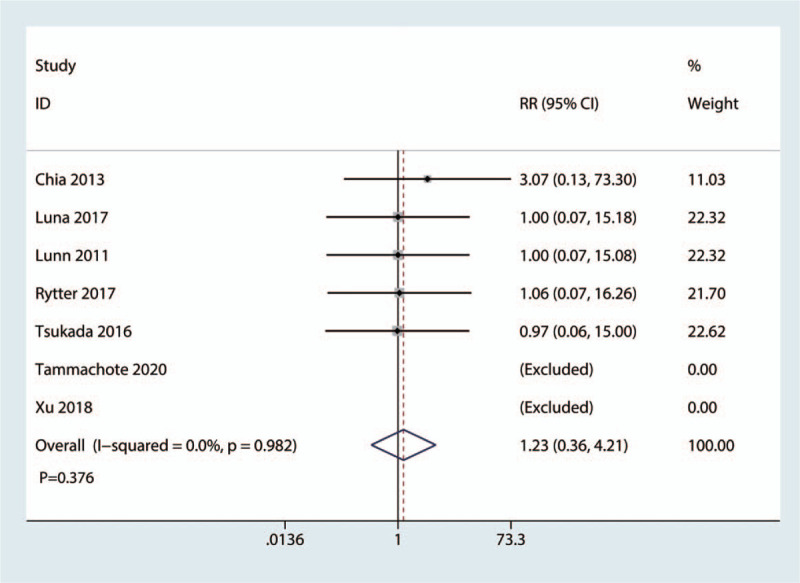
Forest plot of the comparison of periprosthetic knee infection between corticosteroid and control group.

Seven studies with 455 patients recorded the periprosthetic knee infection. No significant differences were found between the corticosteroid group and the control group (RR = 1.23, 95%CI: [0.36, 4.21], *P* = .376; Fig. 7). The fixed-effects model was used to calculate the RR due to the no heterogeneity (*I*^2^ = 0.0%, *P*_heterogeneity_ = .376; Fig. 7).

#### 3.3.5 Length of stay

**Figure 8:**
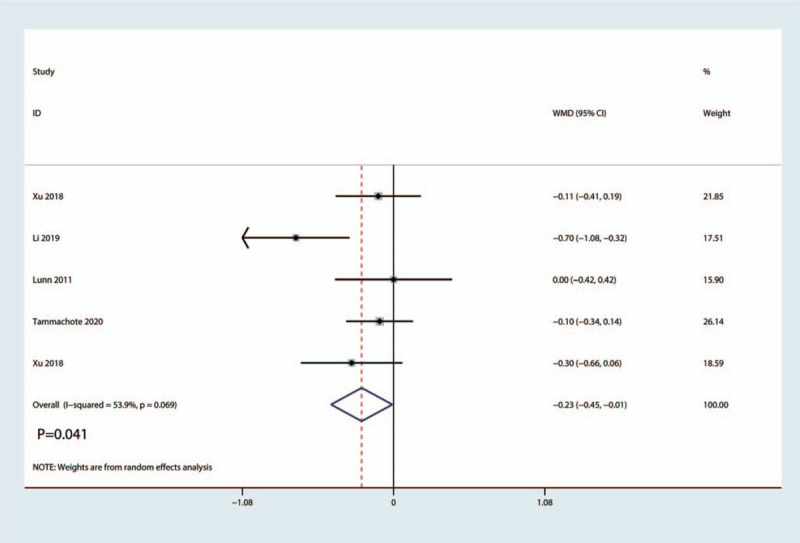
Forest plot of the comparison of length of hospital stay between corticosteroid and control group.

Five studies totaling 412 patients reported data about the length of hospital stay. Pooled data indicated that the corticosteroid group was associated with a significantly reduction of the length of hospital stay (WMD = −0.23, 95%CI: [−0.45, −0.11], *P* = .041; Fig. 8). We used a random effect model due to the statistical heterogeneity (*I*^2^ = 53.9%, *P*_heterogeneity_ = .069, Fig. 8).

#### 3.3.6 The occurrence of nausea

**Figure 9:**
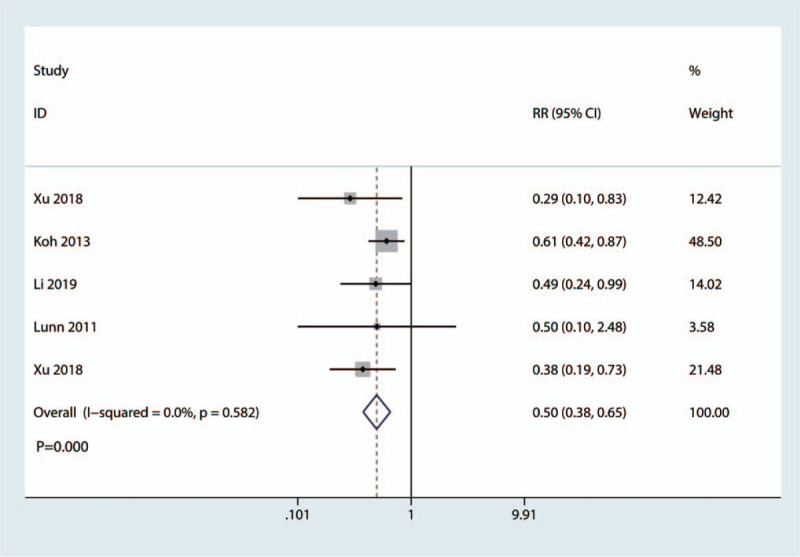
Forest plot of the comparison of the occurrence of nausea between corticosteroid and control group.

Five studies totaling 412 patients investigated the occurrence of nausea. Pooled data indicated that the corticosteroid group was associated with a significantly reduction of the occurrence of nausea (RR = 0.50, 95%CI: [0.38, 0.65], *P* = .000; Fig. 9). We used a fixed effect model due to the low statistical heterogeneity (*I*^2^ = 0.0%, *P*_heterogeneity_ = .582, Fig. 9).

#### 3.3.7 CRP

**Figure 10:**
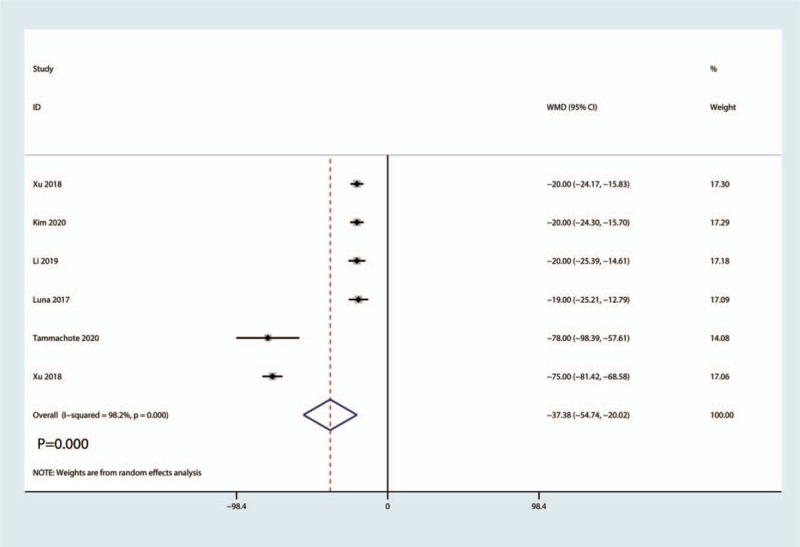
Forest plot of the comparison of the CRP level between corticosteroid and control group.

Six studies totaling 465 patients reported data about the CRP level. Pooled data indicated that the corticosteroid group was associated with a significantly reduction of the CRP level (MD = −37.38, 95%CI: [−54.74, −20.02], *P* = .000; Fig. 10). We used a random effect model due to the high statistical heterogeneity (*I*^2^ = 98.2%, *P*_heterogeneity_ = .000, Fig. 10).

#### 3.3.8 Sensitivity analysis

**Figure 11:**
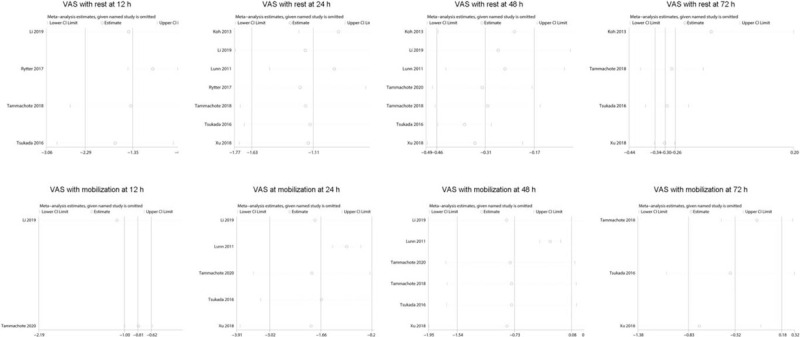
Sensitivity analysis for VAS with rest or mobilization at 12 hour, 24 hour, 48 hour and 72 hour between corticosteroid and control group.

The sensitivity analyses were performed by omitting one study at a time to gauge the robustness of our results. In the sensitivity analysis of VAS with rest at 72 hour (Fig. 11), the influence of each study on the pooled WMD was changed by after excluding the study of Koh et al For VAS with mobilization at 12 hour, after omitting the study by Li et al but were in general similar.

## 4 Discussion

This is the first meta-analysis with maximum sample size to our knowledge conducting the analgesic effect of corticosteroid following TKA. Our pooled data indicated that the corticosteroid was more effective than the control group in terms of VAS at rest and movement, and total morphine consumption.

There are at least 3 potential strength of this meta-analysis. First, this meta-analysis is the first meta-analysis with 1287 subjects that provide quantitative estimates of corticosteroid for pain control in TKA. Second, included studies were limited to RCT design which promote drawing stable results. Third, this meta-analysis has led to specific recommendation for administration glucocorticoids for pain control after TKA.

There are still controversies about the use of corticosteroid following TKA. Luna et al^[15]^ indicated that no differences in proportion of patients with moderate to severe pain was found between the corticosteroid and placebo groups at 24 hours or at 48 hours, and no difference between 2 groups in postoperative sensitization. However, Tammachote et al^[23]^ found that intravenous dexamethasone relieves postoperative pain between 12 hours to 21 hours after TKA and may be a useful adjunct for controlling pain in patients undergoing TKA.

This meta-analysis was conducted as findings on the effects of corticosteroid on pain control after TKA. VAS is a very important index of postoperative pain. VAS was the primary outcome assessed in our meta-analysis. VAS can comprehensively assess the pain of patient after TKA. Our pooled data showed that corticosteroid was better for postoperative pain relieve in patients with TKA.

In our meta-analysis, we demonstrated that corticosteroid group got better VAS at rest and movement. Similar findings were reported by Koh et al^[13]^ and Tsukada et al.^[24]^ Therefore, we conducted compared with the control group, the corticosteroid group provided better analgesic effects for patients undergoing TKA.

Total morphine equivalent consumption was also important post-operative indicators to evaluate the analgesic effects. A randomized controlled trail conducted by Kardash et al^[25]^ reported that the control group is comparable to the corticosteroid group in terms of total equivalent morphine consumption. Luna et al^[15]^ also reported no significant differences between 2 groups in total equivalent morphine consumption. Recently published studies represented different ideas. Samona et al^[18]^ reported patients who received corticosteroids required a significant smaller quantity of oral opioids than that of the control group. Koh et al^[13]^ also revealed that corticosteroid pre-treatment was associated with a reduction of the morphine consumption.

The results of our meta-analysis are in consensus of the recent findings. Pooled data indicated that corticosteroid group consume less opioids compared to control group. We also pooled the data of periprosthetic knee infection. Pooled data showed that no significant differences were found between the corticosteroid group and control group. Length of postoperative stay results from the studies included in this meta-analysis showed that corticosteroid in TKA significantly reduced length of postoperative stay as compared with control group by 0.23 days. Klement et al^[26]^ found that postoperative hospital stay was associated with venous thromboembolic events in the multivariate analysis.

CRP level between corticosteroid and control group was also compared to reflect the inflammation. As expected, corticosteroid could significantly decrease the CRP level. This can also explain corticosteroid may through regulates inflammation and therefore relieve pain.

Also, there are some limitations in our meta-analysis. First, only 13 studies in our meta-analysis. The test power for statistical would be more credible if more RCT are included. Second, unavoidable heterogeneity (samples, country, anesthesia methods, age, and so on) between the included studies may affect the results of pooled data. Third, there was certain heterogeneity between various studies for final outcomes such as VAS with rest or mobilization. However, we did not find the source of heterogeneity through a sensitivity analysis. Although some limitations exist in our study, high quality of included studies and accurate statistical method ensured the reliability of our meta-analysis.

## 5 Conclusions

In conclusion, corticosteroid administration was effective for reducing postoperative pain and morphine consumption after TKA. More important, corticosteroid can reduce the length of hospital stay, the occurrence of nausea without increasing the risk of periprosthetic knee infection when compared to the control group. Well-designed studies with large-size sample are necessary in the future to validate the optimal dosage of and type of corticosteroid in this present meta-analysis.

## Acknowledgments

We thank the authors of the included studies for their help.

## Author contributions

JZ and JXH contributed to the conception and design of the study. JXH performed the statistical analysis and drafted the manuscript. JZ and JXH contributed to the literature search and study selection. JZ and JXH contributed to the quality assessment. JZ and JXH contributed to the data extraction. JZ contributed to the revisions of the manuscript. All authors read and approved the final manuscript.

**Data curation:** Ji-xun Huang.

**Investigation:** Ji-xun Huang.

**Resources:** Jiao Zhang.

**Software:** Jiao Zhang.

**Writing – original draft:** Ji-xun Huang.

**Writing – review and editing:** Ji-xun Huang.
